# Angioedema due to Systemic Isotretinoin Therapy

**DOI:** 10.1155/2014/595914

**Published:** 2014-12-23

**Authors:** Pelin Üstüner

**Affiliations:** Dermatology Clinic, Rize State Hospital, Eminettin Mahallesi, 53100 Rize, Turkey

## Abstract

Angioedema is the swelling of the mucosal membranes as a variant of urticaria induced by hereditary C1 esterase inhibitor enzyme deficiency, certain foods, or drugs. Herein, we report the case of a 23-year-old woman, with mild-moderate acne presenting with widespread facial angioedema on the 2nd day of systemic isotretinoin treatment. The patient had taken no drugs other than isotretinoin in the preceding days and had no known food allergy. Her angioedema was resolved after the isotretinoin was discontinued. We want to draw the attention of dermatologists to this rare adverse allergic effect of isotretinoin which is frequently used in the treatment of acne vulgaris.

## 1. Introduction

Retinoids are widely used to treat many conditions; in particular, isotretinoin is known as the first choice treatment for nodulocystic acne vulgaris that is unresponsive to conventional therapies [1]. While cheilitis, mucositis, xerophthalmia, xerosis, retinoid dermatitis, dyslipidemia, photosensitivity, pyogenic granuloma, onycholysis, and paronychia are the well-known, frequent adverse effects of isotretinoin [2], angioedema due to the use of isotretinoin has only been reported in 3 cases to date [3–5].

Angioedema is the swelling of mucosal membranes as a variant of urticaria which can be induced by hereditary C1 esterase inhibitor enzyme deficiency, certain foods, or drugs [6]. Angioedema may have many causes such as nonsteroidal anti-inflammatory drugs, angiotensin-converting enzyme inhibitors, radiocontrast media, antibiotics, or sea food. Angioedema consists of an allergic (IgE-mediated) or nonallergic hypersensitivity reaction, such as pseudoallergy or idiosyncrasy [6]. Skin tests and determination of specific IgE antibodies with standardized allergens are available, but the pathogenesis of drug-induced urticaria and angioedema is rarely clear. It can involve an allergic (IgE-mediated) or nonallergic hypersensitivity reaction, both with a similar clinical presentation.

## 2. Case Report

**Figure 1:**
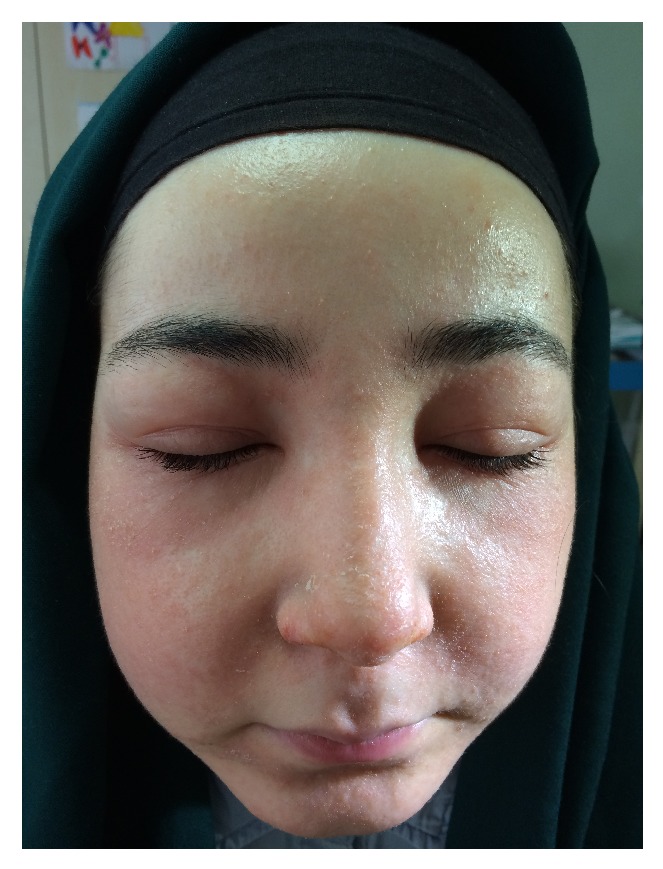
Bilateral periorbital edema, facial widespread erythema, and mild desquamation.

A 23-year-old white woman with mild-moderate acne was referred to our dermatology department with facial recalcitrant acne of 2 years duration. She was unresponsive to previous tetracycline treatment (1000 mg daily) for 1 year and was started on oral isotretinoin. After taking just two doses of 30 mg/day isotretinoin, the patient presented the next day with widespread facial swelling predominant in the periorbital areas. She had no previous history of urticaria or angioedema and no known food allergy. She was on a normal diet and had taken no drugs other than isotretinoin in the preceding days. Her medical history and systemic medication were unremarkable. She had no other symptoms. The results of the blood tests including C1 esterase inhibitor, ASO, hepatitis markers, HIV, and parasitological tests were normal. She stated that she had no prior infectious diseases, contact allergic reactions, or food allergies. Dermatological examination revealed bilateral periorbital oedema, widespread facial erythema, and mild desquamation (Figure 1). No other parts of the body were involved. We diagnosed angioedema due to isotretinoin use, and a systemic steroid 40 mg prednol IM for 3 sequential days and oral levocetirizine 5 mg were initiated. Before the occurrence of the angioedema the patient also had mild seborrhoeic dermatitis. The angioedema disappeared completely in a few days, and the treatment was discontinued.

The patient was rehospitalized for the oral provocation test with 30 mg/day isotretinoin treatment. Vasoactive drugs for cardiopulmonary resuscitation (adrenaline and atropine), methylprednisolone, pheniramine maleate ampules, and tracheal intubation were all prepared before the drug administration in case of an angioedema attack and anaphylactic shock. After reexamination of the similar facial oedema and periorbital swelling that appeared in a few hours, systemic steroid treatment was restarted and angioedema was finally reameliorated. In the following 12 months she had no relapses.

## 3. Discussion

We considered that certain drugs might have been recently started before the occurrence of the angioedema; however, the patient was receiving no drugs apart from isotretinoin. Due to the lack of treatment alternatives and the tendency for the patient to develop scars, an oral provocation test was administered ten days after she was rehospitalized and restarted on isotretinoin therapy. We verified that isotretinoin caused angioedema in this case. Similar facial oedema and periorbital swelling were reinduced on the first day of the second cure. Systemic steroid treatment was restarted and angioedema was finally reameliorated. In the following 12 months she had no relapses.

The possible reasons for this patient's facial swelling include some type of retinoid induced angioedema, exacerbation of inflammation by isotretinoin, and isotretinoin induced capillary leak syndrome [4]. In this case, the etiology of angioedema was evaluated and other common causes, such as food and drug allergies, were eliminated since there was no history of suspicious food intake at the time the lesions developed and the patient was taking no other medications. Furthermore, the facial angioedema appeared shortly after the isotretinoin intake, disappeared after the drug was discontinued, and recurred after it was restarted. In addition, there were no later recurrences of angioedema. Therefore, we were able to confirm the exact etiology of the angioedema as isotretinoin. The desquamation was also attributed to the fact that she also had mild seborrhoeic dermatitis before the occurrence of angioedema, since there was no suspicious contact history and the desquamation was predominantly common in seborrheic areas.

Angioedema has only been reported in 3 cases due to the use of isotretinoin and in 1 case due to the use of acitretin to date [3–5] (Table 1). In one of these cases, both urticaria and angioedema were reported due to systemic isotretinoin treatment in an 18-year-old female referred with facial nodulocystic acne of two years duration [5]. Oedema of the lips and periorbital region and urticarial plaques on the patient's knees 1 day after isotretinoin reintake were revealed. Upon questioning, the patient admitted that she had restarted the isotretinoin treatment on her own the day before [5]. Lip swelling due to the intake of systemic isotretinoin has also been reported in a 15-year-old boy presenting with a severe lip abscess requiring incision and drainage and hospital admission for the application of an intravenous antibiotic [7]. Although rare, lip abscesses related to isotretinoin therapy present with substantial morbidity and should be promptly recognized. Misdiagnosis of mucositis and angioedema may delay the administration of appropriate therapy. Furthermore, perioral abscess formation in patients taking isotretinoin may also masquerade as angioedema or severe mucositis [8]. However, to date only one case of a 48-year-old man with bilateral periorbital oedema related to acitretin 25 mg/day use has been reported [3].

Isotretinoin capsules contain several additives, such as butylated hydroxyanisole, parabens, vegetable oils, and dyes. It is possible that one of these additives might have been the cause of the angioedema [5]. Gelatine capsules contain glycerine and parabens (methyl and propyl), with the following dye systems: 10 mg iron oxide (red) and titanium dioxide; 20 mg FD&C Red No. 3, FD&C Blue No. 1, and titanium dioxide; 40 mg FD&C Yellow No. 6, D&C Yellow No. 10, and La Roche-type titanium dioxide [3]. However, these additives in drugs and foods have not been found to produce angioedema [3]. We were also able to exclude the possible diagnosis of soybean, peanut, titanium dioxide, or iron oxide dermatitis after the application of the allergy prick test with these ingredients, as they are known to be also included in the isotretinoin capsules.

We think that some angioedema cases might have been misdiagnosed, since isotretinoin is not known to have any allergic side effects. Since isotretinoin is frequently used, we believe that dermatologists should be aware of this rare adverse effect of this drug. Furthermore, more clinical and experimental studies should be undertaken to determine the exact association between isotretinoin and angioedema.

## Figures and Tables

**Table 1:** Brief summary of the cases of angioedema due to the treatment of isotretinoin.

Cases	Age	Sex	Clinical features
Saray and Seçkin [5].	18	F	First episode: oedema of the lips, eyelids, and periorbital region. Second episode: erythematous and oedematous plaques on the dorsum of hands and knees

Filho et al. [3].	24	F	Lip oedema
	48	M	Bilateral periorbital oedema^*^

Scheinfeld and Bangalore [4].	32	M	Facial swelling

The presented case.	23	F	Widespread facial swelling, periorbital oedema, facial erythema, and mild desquamation Oral provocation test: facial oedema and periorbital swelling

^*^The case treated with acitretin.
